# Normative value of upper extremity Y balance test in healthy subjects aged between 18 and 36 years from South India: A cross-sectional study

**DOI:** 10.1371/journal.pone.0335443

**Published:** 2025-10-27

**Authors:** Arjun Pavithran, S. Rajasekar, Joshua Cleland, Varunkumar Ramkumar, Animesh Hazari

**Affiliations:** 1 Institute of Physiotherapy, Srinivas University, City Campus, Mangaluru, India; 2 College of Health Sciences, Gulf Medical University, Ajman, United Arab Emirates; 3 Department of Rehabilitation Sciences, Doctor of Physical Therapy, Program, Tufts University School of Medicine, Boston, United States of America; 4 Department of Physical Medicine and Rehabilitation, Madurai Medical College, Madurai, India; SPRINT - Sport Physical Activity and Health Research & Innovation Center, PORTUGAL

## Abstract

**Introduction:**

The Upper Quarter Y Balance Test (UQYBT) is a validated tool used to assess unilateral upper extremity function in a closed kinetic chain. It evaluates parameters such as mobility, stability, and injury risk, and can inform rehabilitation planning. Normative data have been well established for adolescent and active adult populations in the United States. This study aims to establish normative UQYBT values for healthy adults aged 18–36 years in the Indian subcontinent.

**Methods:**

A total of 190 healthy young adults (95 males and 95 females) aged 18–36 years who met the eligibility criteria were included. Baseline demographic data—age, height, weight, body mass index (BMI), and limb length—were collected. Participants performed the UQ-YBT by reaching in the inferolateral, medial, and superolateral directions using the three-reach box. Average reach distances were calculated for each limb, and composite scores (CS) were normalized using arm length.

**Results:**

Age- and sex-specific reference values were established for both upper extremities and for average bilateral performance. Males demonstrated significantly greater reach distances than females; however, the difference decreased after normalization for limb length. Age-related variations were observed, with participants aged 26–30 years achieving the highest normalized reach scores (p < 0.05).

**Conclusion:**

The age- and sex-specific normative values obtained from this study can serve as benchmarks for assessing shoulder mobility and stability among healthy Indian adults aged 18–36 years.

## Introduction

Shoulder pain is the third most common musculoskeletal complaint presenting in primary care [1]. The shoulder’s structural and functional complexity, characterized by a wide range of motion at the expense of joint stability, makes it susceptible to dislocations and overuse injuries [2]. Young adults and adolescents, particularly those engaged in sports, experience a disproportionately high rate of such injuries [3].

Shoulder injuries may result from cumulative low-energy stress (repetitive with gradual onset), high-energy trauma (acute with rapid onset), or a combination of both (repetitive with sudden onset) [4]. These injuries can vary in severity and may lead to functional impairment or absence from sport. Additionally, dysfunction in surrounding structures, such as the cervicothoracic spine, ribs, and muscles controlling scapular movement, can influence glenohumeral mechanics and contribute to shoulder pain [5]. Notably, altered scapular kinematics have been linked to shoulder dysfunction due to their role in coordinating upper limb movement [6].

In recent years, increasing attention has been given to the role of movement quality and neuromuscular control in both injury prevention and rehabilitation. Functional performance tests offer a means of assessing these qualities in a controlled and repeatable manner. These tests are not only useful for identifying current deficits but also serve as screening tools to predict future injury risk in athletic and physically active populations [7]. Among them, the Upper Quarter Y Balance Test (UQ-YBT) has emerged as a reliable and efficient method for evaluating upper extremity function in a closed kinetic chain position [8–10].

The UQ-YBT requires individuals to maintain stability on one upper limb while reaching with the contralateral arm in three directions—medial, inferolateral, and superolateral [11]. Reach distances are recorded and normalized to limb length, allowing for meaningful comparisons across individuals of different body sizes [12]. The test has been used to explore how variables such as sex, age, limb dominance, and training background affect upper limb performance [13,14]. Normative values for the UQ-YBT have been established in Western, adolescent, and athletic populations, but such data remain scarce for South Asian or Indian cohorts [14,15]. Given the anthropometric, cultural, and physical activity differences across populations, localized norms are crucial for accurate assessment. The UQ-YBT is also useful in identifying asymmetries or movement dysfunctions that may not be apparent during routine clinical assessment [15].

However, while reference values for the UQ-YBT exist for certain populations, there is a lack of normative data for young, healthy Indian adults. Such data are essential for clinicians and researchers seeking to interpret performance and identify deviations that may warrant intervention. Establishing such normative values is essential for informed clinical decision-making, particularly in the contexts of sports rehabilitation, injury risk screening, and return-to-play planning. Furthermore, the early detection of asymmetries or functional deficits may help prevent the progression of overuse injuries, especially in overhead athletes. We hypothesized that males would demonstrate greater UQYBT reach distances than females, and performance would vary by age, with young adults in their mid-20s exhibiting peak scores. Therefore, this study aimed to establish normative values for the UQ-YBT in healthy individuals aged 18–36 years in the Indian subcontinent.

## Methods

### Study design and settings

This cross-sectional observational study was conducted at the Institute of Physiotherapy, Srinivas University, City Campus, Mangaluru, Karnataka, India.

### Ethical approval and consent for participation

**Fig 1:**
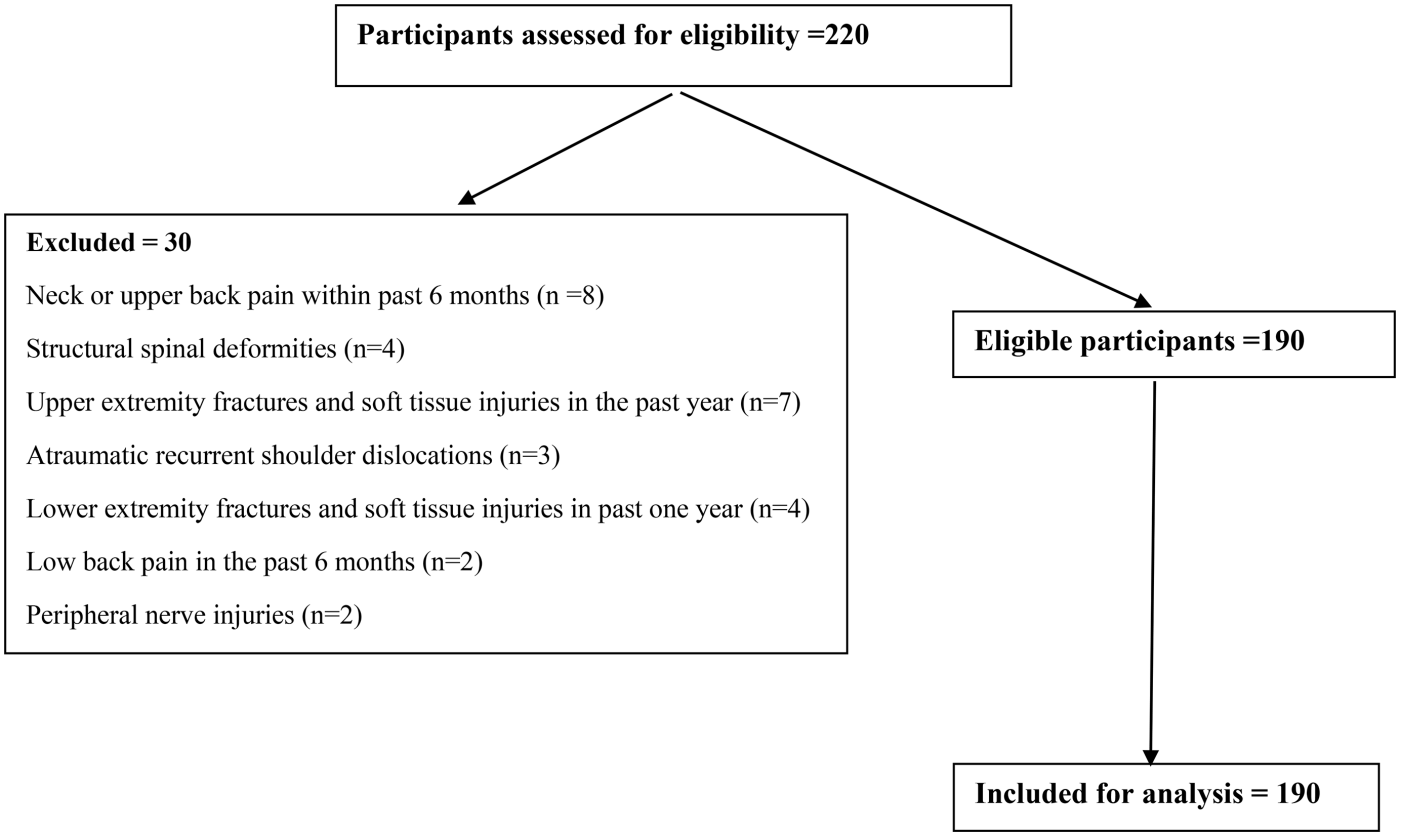
Flow diagram for participants’ inclusion, exclusion, and data analysis samples.

The ethical approval was obtained from the Institutional Ethics Committee, Institute of Physiotherapy, Srinivas University, City Campus, Mangaluru, Karnataka, India (Approval No. SUIP/PG22/130/2022). All participants provided written informed consent before the data collection. Fig 1 illustrates the study’s flow, showing the number of participants screened, excluded, and included for data analysis.

### Sample size

The required sample size was calculated using the formula for estimating a mean:

N = (Zα × σ/ D)² where Zα = 1.96 (for 95% confidence), σ = 21 (pooled standard deviation from prior research) [11], and D = 3 (acceptable margin of error). This yielded an estimated sample size of approximately 188. A final sample size of 190 participants was used.

### Eligibility criteria

Participant eligibility was determined through a set of predefined criteria. The inclusion criteria comprised males and females aged between 18 and 36 years who were identified as young, active adults based on the International Physical Activity Questionnaire (IPAQ) [16]. Exclusion criteria included the presence of neck or upper back pain within the past six months, structural spinal deformities such as scoliosis, a history of soft tissue injury or fracture of the upper extremity within the past year, recurrent shoulder dislocations, a history of soft tissue injury or fracture in the lower extremity, low back pain within the previous six months, and any known neurological conditions.

### Data collection procedure

Participants were classified based on age, sex, Body Mass Index (BMI), and physical activity level as assessed using the IPAQ. The IPAQ is a validated tool used to measure health-related physical activity levels [16,17]. For analysis, participants were divided into five age-based groups, each with a four-year class interval, except for the last group, which comprised a three-year class interval as per the set upper age eligibility criteria.

To ensure consistency and standardisation, the examiner provided all participants with a detailed explanation of the testing procedure. Additionally, the assessors underwent prior training and practiced administering the Upper Quarter Y Balance Test (UQ-YBT) on several individuals before the commencement of actual data collection.

Participants were familiarized with the testing procedure. Testers underwent training and practiced the Upper Quarter Y Balance Test (UQ-YBT) on multiple individuals before data collection to ensure reliability. Before testing, the length of each upper limb was measured separately from the spinous process of C7 to the tip of the middle finger with the shoulder abducted to 90°. A standardized warm-up, including multidirectional shoulder movements, was completed.

**Fig 2:**
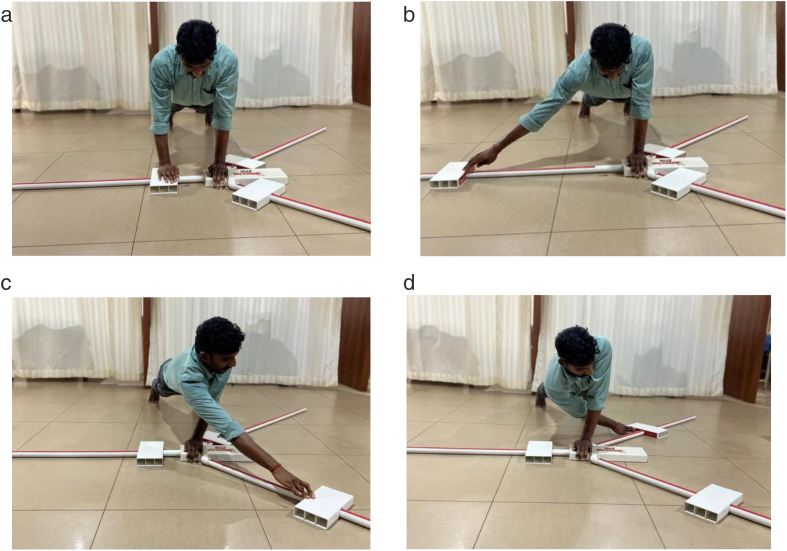
a: Starting position. b: Medial direction (MD). c: Suprolateral direction (SL). d: Inferolateral direction (IL).

The Y Balance Tool (Functional Movement Systems™) was used to perform the test. This tool features a stance platform with three rods positioned at specific angles forming a “Y” to allow measurement in the medial (MD), inferolateral (IL), and superolateral (SL) directions. Participants assumed a push-up position with feet shoulder-width apart (Fig 2a) and performed four practice attempts on each side. For the actual test, participants reached in the three specified directions using the non-weight-bearing arm while maintaining balance on the opposite arm. The medial direction reach is shown in Fig 2b, the superolateral direction in Fig 2c, and the inferolateral direction in Fig 2d. The reaching arm was not allowed to touch the ground, and the participant had to return to the starting position to complete the movement. Each direction was reached once per trial, and three trials were performed for each arm. A 30-second rest was given between trials to reduce fatigue and ensure consistent performance [18]. Participants then switched sides, performing the same procedure with the opposite arm as the weight-bearing limb. Reach distances were measured in centimetres, and three values per direction were recorded. The average of the three attempts in each direction was used for analysis.

### Data normalization and reporting

The data was normalized to limb length as given below:


Normalized score = (Reach Distance ÷ Limb Length) × 100.

Composite Score = (Sum of three normalized reach distances ÷ 3 × Limb Length) × 100.

The study adheres to all strengthening the Reporting of Observational Studies in Epidemiology (STROBE) guidelines and presents the required data accordingly.

### Data analysis

Normality of the data was assessed using the Kolmogorov–Smirnov test. As the data were not normally distributed, descriptive statistics are reported as medians with interquartile ranges (IQR). Age-group comparisons were analysed using the Kruskal–Wallis test. The Mann–Whitney U test was used to examine differences between males and females in test scores. Statistical significance was set at p ≤ 0.05.

## Results

**Fig 3:**
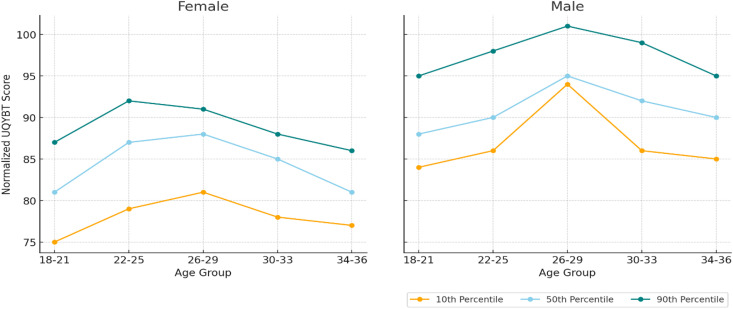
Percentile values and curves of normalized UQYBT by sex and age group.

A total of 190 participants (95 males and 95 females) aged 18–36 years were included. Descriptive demographic characteristics and between-sex comparisons are presented in Table 1. Statistically significant differences were observed between males and females in height, weight, BMI, and upper limb length (p < 0.001). Sex-specific percentile values and curves (10th, 50th, and 90th percentile) is presented in Fig 3.

**Table 1:** Demographic dimensions of male (n = 95) and female (n = 95) recruited (Median and Inter-Quartile Range is shown).

Demographic dimensions	Total (n = 190)	Male (n = 95)	Female (n = 95)	p-value*
Age (Years)	26.4 (26.2 to 27.8)	26.4 (25.9 to 28.1)	26.4 (25.9 to 28.1)	>.999
Height (cm)	167.1 (166 to 168.6)	172.7 (171.6 to 174.1)	161.6 (160.2 to 163.4)	<.001
Weight (kg)	62.6 (62.1 to 66)	69.4 (68.8 to 74.7)	53.9 (53.4 to 57.6)	<.001
BMI (kg/m^2^)	22.4 (22.1 to 23.2)	23.7 (23.3 to 24.7)	21.1 (20.7 to 21.9)	<.001
UL length – R (cm)	82.9 (82.3 to 84.1)	87.3 (86.5 to 88.1)	78.9 (78.1 to 80.2)	<.001
UL length – L (cm)	82.9 (82.3 to 84.1)	87.3 (86.5 to 88.1)	78.9 (78.1 to 80.2)	<.001

*- Statistical significance between male and female reported by Mann-Whitney U test

### Raw reach distances

As shown in Table 2, males demonstrated significantly greater absolute reach distances than females across all three directions, medial (MD), superolateral (SL), and inferolateral (IL),as well as higher composite scores (CS). For example, in the dominant arm, median MD reach was 77.5 cm (IQR: X–X) in males versus 70.2 cm (IQR: X–X) in females (p < 0.001). Similar trends were observed for SL and IL directions on both arms.

**Table 2:** Overall and sex specific Mean reach distance of UQ-YBT (Median and Inter-Quartile Range is shown).

Variables	Total (n = 190)	Male (n = 95)	Female (n = 95)	p-value*
Reach distance (cm)				
Medial (cm)				
Right	77.5 (76.7 to 79.7)	83.0 (81.6 to 85.8)	72.4 (71.2 to 74.3)	<.001
Left	76.4 (75.6 to 78.5)	82.1 (80.7 to 84.7)	71.1 (69.9 to 72.9)	<.001
Inferolateral (cm)				
Right	63.3 (62.6 to 65.5)	68.7 (67.5 to 71.3)	58.2.5 (57.1 to 60.4)	<.001
Left	62.7 (61.9 to 65.3)	67.2 (65.8 to 70.5)	58.4 (57.2 to 61.1)	<.001
Superolateral (cm)				
Right	54.8 (54.2 to 57.2)	58.9 (57.7 to 62.1)	50.9 (49.9 to 53)	<.001
Left	53.8 (53.2 to 56.2)	58.6 (57.4 to 61.6)	49.3 (48.3 to 51.7)	<.001
Composite (cm)**				
Right	215.9 (213.8 to 220.4)	230.0 (226.7 to 235.2)	202.7 (200.0 to 206.6)	<.001
Left	213.8 (211.7 to 218.3)	228.7 (225.6 to 233.6)	199.9 (197.2 to 203.8)	<.001

*- Statistical significance between male and female reported by Mann-Whitney

** sum of the 3-reach distance (medial, superolateral, inferolateral)

### Normalized reach distances

When reach distances were normalized to limb length (Table 3), males continued to outperform females in most directions. However, the sex difference was attenuated, particularly for the medial direction of the dominant arm, where normalized scores were comparable (p = ns). Composite scores remained significantly higher in males after normalization (p < 0.05).

**Table 3:** Overall and sex specific Normalized Mean reach distance of UQ-YBT (Median and Inter-Quartile Range is shown).

Variables	Total (n = 190)	Male (n = 95)	Female (n = 95)	p-value*
Normalized Reach Distance (cm)†				
Medial				
Right	93.4 (92.4 to 95.6)	95.1 (93.5 to 98.2)	91.7 (90.1 to 94.3)	.011
Left	92.0 (91.1 to 93.9)	94.1 (92.6 to 96.8)	90.0 (88.7 to 92.2)	<.001
Inferolateral				
Right	76.2 (75.4 to 78.5)	78.8 (77.3 to 81.8)	73.7 (72.4 to 76.3)	<.001
Left	75.5 (74.7 to 78.1)	77.0 (75.5 to 80.7)	73.9 (72.5 to 76.9)	.027
Superolateral				
Right	65.9 (65.3 to 68.5)	67.6 (66.1 to 71.2)	64.5 (63.2 to 67.1)	.041
Left	64.8 (64.1 to 67.3)	67.2 (65.8 to 70.6)	62.5 (61.2 to 65.3)	.004
Composite‡				
Right	78.7 (77.9 to 80.7)	80.7 (79.2 to 83.5)	76.8 (75.6 to 78.9)	.004
Left	77.7 (76.9 to 79.6)	79.7 (78.2 to 82.4)	75.7 (74.5 to 77.8)	.002

*- Statistical significance between male and female reported by Mann-Whitney

†Reach distance divided by limb length multiplied by 100

‡Sum of the 3 normalized reach distances (medial, supralateral, inferolateral) divided by 3 times limb length multiplied by 100

### Age-based differences

The reach performance stratified by age groups is presented in Tables 4 and 5. Participants in the 26–30-year age group generally achieved the highest reach distances in both raw and normalized scores. Statistically significant differences were found across age groups in the superolateral and inferolateral directions (p < 0.05), suggesting an age-related variation in upper extremity dynamic stability and control. Table 6 indicates side-to-side asymmetry values per reach direction.

**Table 4:** Mean reach distance of UQ-YBT stratifying for age (Median and Inter-Quartile Range is shown).

Variables	18-21 years (n = 40)	22-25 years (n = 40)	26-29 years (n = 40)	30-33 years (n = 40)	34-36 years (n = 30)	p-value*
Reach distance (cm)						
Medial						
Right	77.3 (73.6 to 80.8)	77.0 (74.5 to 80.7)	79.9 (77.2 to 84.4)	76.3 (73.4 to 81.1)	76.7 (73.6 to 80.8)	.454
Left	75.4 (71.8 to 79.2)	77.6 (75.0 to 81.2)	79.9 (77.4 to 83.5)	73.9 (71.2 to 78.2)	74.9 (71.8 to 79.2)	.060
Inferolateral						
Right	63.7 (58.9 to 65.3)	63.3 (60.9 to 67.0)	65.1 (62.6 to 69.3)	61.9 (59.4 to 66.1)	61.9 (58.9 to 66.3)	.457
Left	66.1 (56.9 to 64.6)	63.1 (60.7 to 66.8)	66.0 (63.0 to 71.5)	57.9 (55.5 to 61.6)	60.0 (56.9 to 64.6)	.004
Superolateral						
Right	56.2 (49.4 to 55.8)	54.1 (51.5 to 58.6)	56.7 (54.4 to 60.6)	54.3 (51.7 to 58.9)	51.9 (49.4 to 55.8)	.209
Left	57.2 (48.7 to 55.4)	54.1 (51.6 to 58.5)	55.1 (52.7 to 59.6)	50.7 (48.3 to 55.1)	51.3 (48.7 to 55.4)	.033
Composite						
Right	217.7 (203.9 to 219.6)	216.4 (210.4 to 224.5)	219.9 (213.3 to 229.2)	213.7 (206.9 to 223.5)	210.8 (203.9 to 219.6)	.387
Left	216.7 (201.1 to 217.8)	216.9 (210.9 to 225.1)	218.3 (211.9 to 227.2)	207.6 (201.1 to 216.9)	208.3 (201.1 to 217.8)	.114

**Note:** Demographic dimensions do not follow a normal distribution. Hence, is expressed as the geometric mean with a 95% confidence interval. *- Statistical significance between different age groups reported by Kruskal-Wallis Test

**Table 5:** Mean reach distance of UQ-YBT stratifying for age.

Variables	18-21 years (n = 40)	22-25 years (n = 40)	26-29 years (n = 40)	30-33 years (n = 40)	34-36 years (n = 30)	p-value*
Normalized Reach Distance (cm)						
Medial						
Right	92.7 (90.9 to 97.7)	91.0 (88.2 to 95.1)	96.6 (93.7 to 101.1)	92.7 (89.8 to 97.0)	93.9 (90.9 to 97.7)	.167
Left	90.5 (88.8 to 95.3)	91.7 (89.0 to 95.4)	96.6 (94.2 to 99.9)	89.8 (87.3 to 93.2)	91.7 (88.8 to 95.3)	.016
Inferolateral						
Right	76.5 (72.7 to 79.9)	74.8 (72.3 to 78.6)	78.7 (75.9 to 83.2)	75.2 (72.7 to 78.9)	75.7 (72.7 to 79.9)	.435
Left	79.3 (70.3 to 78.0)	74.5 (71.9 to 78.6)	79.9 (76.6 to 85.4)	70.3 (67.9 to 73.7)	73.5 (70.3 to 78.0)	<.001
Superolateral						
Right	67.4 (60.7 to 67.9)	63.9 (61.1 to 68.6)	68.6 (66.1 to 72.4)	63.9 (63.2 to 70.4)	63.6 (60.7 to 67.9)	.195
Left	68.6 (59.8 to 67.3)	63.9 (61.2 to 68.6)	66.7 (63.9 to 71.2)	61.6 (59.1 to 65.8)	62.8 (59.8 to 67.3)	.037
Composite						
Right	79.2 (75.3 to 81.3)	76.8 (74.3 to 80.3)	81.5 (78.8 to 85.3)	78.1 (75.7 to 81.7)	77.9 (75.3 to 81.3)	.161
Left	79.8 (73.6 to 79.6)	76.9 (74.4 to 80.4)	81.2 (78.6 to 85.2)	74.1 (71.9 to 76.9)	76.2 (73.6 to 77.6)	.006

**Note:** Demographic dimensions does not follow normal distribution. Hence, expressed in geometric mean with 95% confidence interval. *- Statistical significance between different age groups reported by Kruskal-Wallis Test

**Table 6:** Normalised side-to-side asymmetry values by age and sex.

Sex	Age Group	Medial Asymmetry (cm)	Superolateral Asymmetry (cm)	Inferolateral Asymmetry (cm)
Male	18–21	1.5 (0.8–2.3)	2.0 (1.2–2.9)	1.8 (1.0–2.5)
Male	22–25	1.4 (0.9–2.1)	1.8 (1.1–2.6)	1.6 (1.0–2.3)
Male	26–29	1.2 (0.7–2.0)	1.6 (0.9–2.4)	1.4 (0.8–2.1)
Male	30–33	1.3 (0.8–2.1)	1.7 (1.0–2.5)	1.5 (0.9–2.3)
Male	34–36	1.4 (0.9–2.2)	1.9 (1.1–2.7)	1.6 (1.0–2.4)
Female	18–21	1.8 (1.0–2.7)	2.2 (1.3–3.2)	2.0 (1.1–2.9)
Female	22–25	1.7 (1.0–2.5)	2.1 (1.2–3.0)	1.9 (1.1–2.8)
Female	26–29	1.6 (0.9–2.4)	2.0 (1.1–2.8)	1.9 (1.0–2.7)
Female	30–33	1.7 (1.0–2.6)	2.2 (1.3–3.1)	2.0 (1.1–2.9)
Female	34–36	1.8 (1.1–2.7)	2.3 (1.4–3.2)	2.1 (1.2–3.0)

## Discussion

This study aimed to establish normative values for the Upper Quarter Y Balance Test (UQYBT) among healthy adults aged 18–36 years in the Indian subcontinent. To our knowledge, this is among the first studies to generate such region-specific data, addressing a notable gap in the literature on functional assessment in South Asian populations. A key strength of this study lies in its balanced male–female ratio and relatively large sample size, which enhances the reliability of sex-based comparisons. Consistent examiner training and standardized protocols further minimized measurement bias. We hypothesized that males would demonstrate greater UQYBT performance and that scores would vary by age, peaking in mid-adulthood. Our findings supported both assumptions. The study confirmed sex- and age-related differences in UQYBT performance, with males demonstrating significantly greater reach distances. The subgroup analysis for age revealed maximum reach distance in the 22–25 age group among females, and the 26–29 age group among males. These differences likely result from physiological and biomechanical factors. Males typically have greater upper-body strength and power [19], due in part to higher testosterone levels, which promote muscular hypertrophy and neuromuscular efficiency. Enhanced shoulder girdle and trunk strength likely contribute to postural control and dynamic stability in the closed kinetic chain position used during the test. Additionally, better proprioceptive acuity [20] and core stability may support multidirectional reaching, explaining the higher performance observed in males.

Our findings are broadly consistent with those reported in U.S. military cohorts. Teyhen et al. (2014) observed that performance on functional movement tasks, including the UQYBT, was significantly influenced by age and sex, with younger service members and males performing better [11]. In our study, the 26–30-year age group demonstrated peak scores, aligning with the notion that neuromuscular efficiency and control are optimal in young adulthood. Schwiertz et al. (2021) established normative UQYBT values for European adolescents and reported substantial variability across age categories, with performance stabilizing in later adolescence [15]. Compared with their data, our South Indian adult cohort showed lower absolute reach distances but similar normalized scores, emphasizing the importance of anthropometric adjustments and region-specific norms. More recently, Steele and Valentin (2024) synthesized evidence from multiple cohorts and highlighted that both intrinsic (e.g., sex, limb dominance) and extrinsic (e.g., sport participation, training background) factors influence UQYBT performance [21]. Our results support this framework, demonstrating sex-related differences that diminish after normalization, as well as age-related trends consistent with physiological development and neuromuscular adaptations. However, these differences diminished after normalization to limb length, aligning with previous U.S.-based studies that reported no significant sex or age differences post-normalization [11].

**Fig 4:**
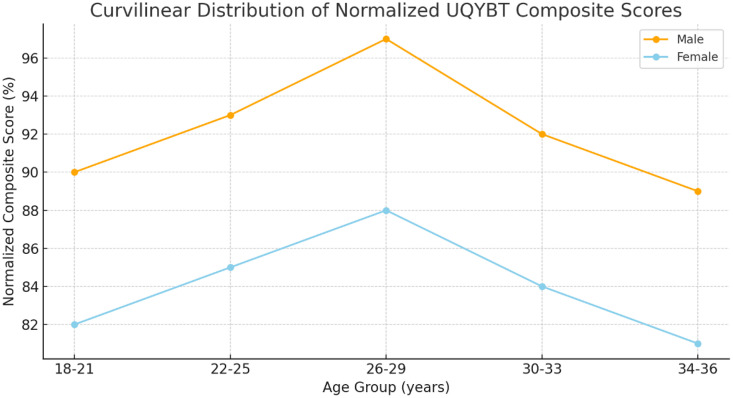
Curvilinear distribution of normalized UQYBT composite score.

In addition, to support our findings, a study reported that age and sex influence UQYBT scores, with older individuals and males generally performing better [21]. Our data also revealed a curvilinear performance distribution, peaking between ages 26 and 29 (Fig 4), possibly reflecting optimal neuromuscular control, proprioception, and mobility during this life stage [22]. Although adolescent scores tend to vary more, this study broadens the understanding of normative values within a stable adult population.

The UQYBT has demonstrated moderate-to-excellent test-retest reliability and low side-to-side asymmetry in healthy adults [23,24]. Our results further validate its utility as a tool for assessing closed-chain upper extremity function. Beyond clinical diagnostics, the UQYBT can aid in injury prevention screening and guide individualized rehabilitation, particularly in overhead sports like swimming, volleyball, and handball where shoulder injuries are prevalent [25–27]. Establishing region-specific normative values improves clinical decision-making and supports more tailored interventions. Together, these findings place our work within a broader international context. While normative UQYBT data from Western cohorts provide useful benchmarks, our study contributes region-specific reference values for South Indian adults, which are necessary for accurate injury risk screening, return-to-play decisions, and rehabilitation planning in this population.

### Clinical Implications

The normative reference values from this study serve as practical benchmarks for physical therapists, athletic trainers, and sports physicians evaluating upper limb function in young adults. They may assist with return-to-sport decisions, early identification of individuals at risk for shoulder dysfunction, and development of sport- or task-specific rehabilitation programs.

## Limitations

This study was conducted at a single center, which may limit generalizability. Larger multicentre studies with a larger sample and more diverse cohorts would further enhance the generalizability of these reference values. The absence of randomized testing sequences may have introduced fatigue-related bias. Furthermore, not controlling for the socioeconomic status of participants, along with the focus on adults aged 18–36 years, limits the applicability of the findings to individuals from different economic backgrounds and to older populations.

## Future directions

Future research should include multicenter trials with more diverse age groups (particularly >40 years), broader ethnic representation, and various athletic populations. Studies should assess test–retest and inter-rater reliability in injured cohorts and explore the predictive value of UQYBT asymmetry in injury prevention. Sport-specific applications also warrant further exploration.

## Conclusion

This study establishes normative UQYBT values in healthy Indian adults aged 18–36 years. The observed sex-specific differences and age-related trends provide important reference data for clinicians and researchers conducting upper extremity assessment. These findings enhance the test’s value in injury screening, rehabilitation planning, and performance optimization in active populations.

## Supporting information

S1 Data (XLSX)

S2 File (DOCX)

## References

[1] Lucas J, van Doorn P, Hegedus E, Lewis J, van der Windt D. A systematic review of the global prevalence and incidence of shoulder pain. BMC Musculoskelet Disord. 2022;23(1):1073. doi: 10.1186/s12891-022-05973-8 36476476 PMC9730650

[2] McCausland C, Sawyer E, Eovaldi BJ, Varacallo M. Anatomy, shoulder and upper limb, shoulder muscles. In: StatPearls. Treasure Island (FL): StatPearls Publishing; 2024. Accessed June 16, 2024 Available from: https://www.ncbi.nlm.nih.gov/books/NBK534836//30521257

[3] Enger M, Skjaker SA, Nordsletten L, Pripp AH, Melhuus K, Moosmayer S, et al. Sports-related acute shoulder injuries in an urban population. BMJ Open Sport Exerc Med. 2019;5(1):e000551. doi: 10.1136/bmjsem-2019-000551 31548901 PMC6733325

[4] Bahr R, Clarsen B, Derman W, Dvorak J, Emery CA, Finch CF, et al. International Olympic Committee consensus statement: methods for recording and reporting of epidemiological data on injury and illness in sport 2020 (including STROBE Extension for Sport Injury and Illness Surveillance (STROBE-SIIS)). Br J Sports Med. 2020;54(7):372–89. doi: 10.1136/bjsports-2019-101969 32071062 PMC7146946

[5] Minkalis AL, Vining RD, Long CR, Hawk C, de Luca K. A systematic review of thrust manipulation for non-surgical shoulder conditions. Chiropr Man Therap. 2017;25:1. doi: 10.1186/s12998-016-0133-8 28070268 PMC5215137

[6] Choi M, Chung J. Biomechanical and functional analysis of the shoulder complex and thoracic spine in patients with subacromial impingement syndrome: a case control study. Medicine (Baltimore). 2023;102(4):e32760. doi: 10.1097/MD.0000000000032760 36705396 PMC9875974

[7] Vaughan B, Theisinger K, Abels L, Bryan L, Duggan S. Normative data and inter-examiner reliability of the upper quarter Y-balance test. Int J Ther Rehabil. 2019;26(6):1–9. doi: 10.12968/ijtr.2018.0020

[8] Liaghat B, Pedersen JR, Husted RS, Pedersen LL, Thorborg K, Juhl CB. Diagnosis, prevention and treatment of common shoulder injuries in sport: grading the evidence - a statement paper commissioned by the Danish Society of Sports Physical Therapy (DSSF). Br J Sports Med. 2023;57(7):408–16. doi: 10.1136/bjsports-2022-105674 36261251 PMC10086287

[9] Teixeira AL, Oliveira AS de, Rodrigues NA, Bueno GAS, Novais MEO, Moreira R de P, et al. Reference values, intrarater reliability, and measurement error for the closed kinetic chain upper extremity stability test and upper quarter y balance test in young adults. Motriz: rev educ fis. 2022;28. doi: 10.1590/s1980-657420220009921

[10] Greenberg ET, Barle M, Glassmann E, Jung M-K. Interrater and test-retest reliability of the Y balance test in healthy, early adolescent female athletes. Int J Sports Phys Ther. 2019;14(2):204–13. doi: 10.26603/ijspt20190204 30997273 PMC6449012

[11] Gorman PP, Butler RJ, Plisky PJ, Kiesel KB. Upper quarter Y Balance Test: reliability and performance comparison between genders in active adults. J Strength Cond Res. 2012;26(11):3043–8. doi: 10.1519/JSC.0b013e3182472fdb 22228174

[12] Teyhen DS, Riebel MA, McArthur DR, Savini M, Jones MJ, Goffar SL, et al. Normative data and the influence of age and gender on power, balance, flexibility, and functional movement in healthy service members. Mil Med. 2014;179(4):413–20. doi: 10.7205/MILMED-D-13-00362 24690966

[13] Schwiertz G, Beurskens R, Muehlbauer T. Discriminative validity of the lower and upper quarter Y balance test performance: a comparison between healthy trained and untrained youth. BMC Sports Sci Med Rehabil. 2020;12(1):73. doi: 10.1186/s13102-020-00220-w 33292443 PMC7713321

[14] Westrick RB, Miller JM, Carow SD, Gerber JP. Exploration of the y-balance test for assessment of upper quarter closed kinetic chain performance. Int J Sports Phys Ther. 2012;7(2):139–47. 22530188 PMC3325634

[15] Schwiertz G, Bauer J, Muehlbauer T. Upper quarter Y Balance Test performance: normative values for healthy youth aged 10 to 17 years. PLoS One. 2021;16(6):e0253144. doi: 10.1371/journal.pone.0253144 34143826 PMC8213051

[16] Clina JG, Sayer RD, Friedman JE, Chui TK, Mehta T, Rimmer JH, et al. Reliability and validity of the International Physical Activity Questionnaire adapted to include adults with physical disability. J Phys Act Health. 2023;21(2):189–96. doi: 10.1123/jpah.2023-0504 38056440 PMC10875625

[17] Sember V, Meh K, Sorić M, Starc G, Rocha P, Jurak G. Validity and reliability of International Physical Activity Questionnaires for adults across EU countries: systematic review and meta analysis. Int J Environ Res Public Health. 2020;17(19):7161. doi: 10.3390/ijerph17197161 33007880 PMC7579664

[18] Hazar Z, Ulug N, Yuksel I. Upper Quarter Y-Balance Test score of patients with shoulder impingement syndrome. Orthop J Sports Med. 2014;2(11_suppl3). doi: 10.1177/2325967114s00275

[19] Kritzer TD, Lang CJ, Holmes MWR, Cudlip AC. Sex differences in strength at the shoulder: a systematic review. PeerJ. 2024;12:e16968. doi: 10.7717/peerj.16968 38525275 PMC10960529

[20] Vafadar AK, Côté JN, Archambault PS. Sex differences in the shoulder joint position sense acuity: a cross-sectional study. BMC Musculoskelet Disord. 2015;16:273. doi: 10.1186/s12891-015-0731-y 26423066 PMC4589903

[21] Steele C, Valentin S. Intrinsic and extrinsic variables impacting Upper Quarter Y Balance Test scores in sporting cohorts: a systematic review. J Bodyw Mov Ther. 2024;39:183–94. doi: 10.1016/j.jbmt.2023.12.010 38876624

[22] Dewolf AH, Sylos-Labini F, Cappellini G, Ivanenko Y, Lacquaniti F. Age-related changes in the neuromuscular control of forward and backward locomotion. PLoS One. 2021;16(2):e0246372. doi: 10.1371/journal.pone.0246372 33596223 PMC7888655

[23] Stanković D, Trajković N, Živković M, Milanović Z. Test–retest reliability of the Upper Quarter Y Balance Test in recreationally active healthy adults. Exerc Qual Life. 2023;15(1):10–6. doi: 10.5937/eqol2315010S

[24] Smajla D, Cvet M, Topič MD, Pišot R, Šarabon N. Upper-extremity physical performance tests in older adults: Reference values, reliability, and measurement error. J Sport Health Sci. 2024;13(2):218–26. doi: 10.1016/j.jshs.2023.07.012 39593514

[25] Sein ML, Walton J, Linklater J, Appleyard R, Kirkbride B, Kuah D, et al. Shoulder pain in elite swimmers: primarily due to swim-volume-induced supraspinatus tendinopathy. Br J Sports Med. 2010;44(2):105–13. doi: 10.1136/bjsm.2008.047282 18463295

[26] Reeser JC, Verhagen E, Briner WW, Askeland TI, Bahr R. Strategies for the prevention of volleyball related injuries. Br J Sports Med. 2006;40(7):594–600; discussion 599-600. doi: 10.1136/bjsm.2005.018234 16799111 PMC2564299

[27] Myklebust G, Hasslan L, Bahr R, Steffen K. High prevalence of shoulder pain among elite Norwegian female handball players. Scand J Med Sci Sports. 2013;23(3):288–94. doi: 10.1111/j.1600-0838.2011.01398.x 22092886

