# Supplementary material for: Normative value of upper extremity Y balance test in healthy subjects aged between 18 and 36 years from South India: A cross-sectional study
Source: PLoS One. 2025 Oct 27;20(10):e0335443. doi: 10.1371/journal.pone.0335443 (PMC12558448; doi:10.1371/journal.pone.0335443)
Supplement: S2 File — (DOCX) [file pone.0335443.s002.docx]

STROBE Statement—Checklist of items that should be included in reports of ***cross-sectional studies***

|  | Item No | Recommendation |
| --- | --- | --- |
| **Title and abstract** | 1 | (*a*) Indicate the study’s design with a commonly used term in the title or the abstract – Line 1 |
|  |  | (*b*) Provide in the abstract an informative and balanced summary of what was done and what was found – Line 28 |
| Introduction | | |
| Background/rationale | 2 | Explain the scientific background and rationale for the investigation being reported – Line 51 |
| Objectives | 3 | State specific objectives, including any prespecified hypotheses – Line 96 |
| Methods | | |
| Study design | 4 | Present key elements of study design early in the paper – Line 99 |
| Setting | 5 | Describe the setting, locations, and relevant dates, including periods of recruitment, exposure, follow-up, and data collection – Line 99 |
| Participants | 6 | (*a*) Give the eligibility criteria, and the sources and methods of selection of participants – Line 114 |
| Variables | 7 | Clearly define all outcomes, exposures, predictors, potential confounders, and effect modifiers. Give diagnostic criteria, if applicable – Line 124 |
| Data sources/ measurement | 8* | For each variable of interest, give sources of data and details of methods of assessment (measurement). Describe comparability of assessment methods if there is more than one group – Line 161 |
| Bias | 9 | Describe any efforts to address potential sources of bias – Line 223 |
| Study size | 10 | Explain how the study size was arrived at – Line 108 |
| Quantitative variables | 11 | Explain how quantitative variables were handled in the analyses. If applicable, describe which groupings were chosen and why – Line 161 |
| Statistical methods | 12 | (*a*) Describe all statistical methods, including those used to control for confounding – Line 161 |
|  |  | (*b*) Describe any methods used to examine subgroups and interactions – Line 161 |
|  |  | (*c*) Explain how missing data were addressed- not applicable |
|  |  | (*d*) If applicable, describe analytical methods taking account of sampling strategy |
|  |  | (*e*) Describe any sensitivity analyses – not applicable |
| Results | | |
| Participants | 13* | (a) Report numbers of individuals at each stage of study—eg numbers potentially eligible, examined for eligibility, confirmed eligible, included in the study, completing follow-up, and analysed- Line 170 |
|  |  | (b) Give reasons for non-participation at each stage |
|  |  | (c) Consider use of a flow diagram |
| Descriptive data | 14* | (a) Give characteristics of study participants (eg demographic, clinical, social) and information on exposures and potential confounders Line 170 |
|  |  | (b) Indicate number of participants with missing data for each variable of interest- Not Applicable |
| Outcome data | 15* | Report numbers of outcome events or summary measures – Line 170 |
| Main results | 16 | (*a*) Give unadjusted estimates and, if applicable, confounder-adjusted estimates and their precision (eg, 95% confidence interval). Make clear which confounders were adjusted for and why they were included |
|  |  | (*b*) Report category boundaries when continuous variables were categorized – Table 1,2,3 |
|  |  | (*c*) If relevant, consider translating estimates of relative risk into absolute risk for a meaningful time period |
| Other analyses | 17 | Report other analyses done—eg analyses of subgroups and interactions, and sensitivity analyses – Table 4 |
| Discussion | | |
| Key results | 18 | Summarise key results with reference to study objectives – Line 215 |
| Limitations | 19 | Discuss limitations of the study, taking into account sources of potential bias or imprecision. Discuss both direction and magnitude of any potential bias – Line 257 |
| Interpretation | 20 | Give a cautious overall interpretation of results considering objectives, limitations, multiplicity of analyses, results from similar studies, and other relevant evidence – Line 268 |
| Generalisability | 21 | Discuss the generalisability (external validity) of the study results – Line 268 |
| Other information | | |
| Funding | 22 | Give the source of funding and the role of the funders for the present study and, if applicable, for the original study on which the present article is based – Line 288 |

*Give information separately for exposed and unexposed groups.

**Note:** An Explanation and Elaboration article discusses each checklist item and gives methodological background and published examples of transparent reporting. The STROBE checklist is best used in conjunction with this article (freely available on the Web sites of PLoS Medicine at http://www.plosmedicine.org/, Annals of Internal Medicine at http://www.annals.org/, and Epidemiology at http://www.epidem.com/). Information on the STROBE Initiative is available at www.strobe-statement.org.
